# Development of Self-Compatible *B. rapa* by RNAi-Mediated *S* Locus Gene Silencing

**DOI:** 10.1371/journal.pone.0049497

**Published:** 2012-11-08

**Authors:** Hee-Jeong Jung, Hyo-Jin Jung, Nasar Uddin Ahmed, Jong-In Park, Kwon-Kyoo Kang, Yoonkang Hur, Yong-Pyo Lim, Ill-Sup Nou

**Affiliations:** 1 Department of Horticulture, Sunchon National University, Suncheon, Republic of Korea; 2 Department of Horticulture, Jeollanam-do Agricultural Research and Extension Services, Najusi, Republic of Korea; 3 Department of Horticulture, Hankyong National University, Ansung, Republic of Korea; 4 Department of Biology, Chungnam National University, Daejeon, Republic of Korea; 5 Department of Horticulture, Chungnam National University, Daejeon, Republic of Korea; University of New England, Australia

## Abstract

The self-incompatibility (SI) system is genetically controlled by a single polymorphic locus known as the *S*-locus in the Brassicaceae. Pollen rejection occurs when the stigma and pollen share the same *S*-haplotype. Recognition of *S*-haplotype specificity has recently been shown to involve at least two S-locus genes, S-receptor kinase (*SRK*) and *S*-locus protein 11 or *S* locus Cysteine-rich (*SP11/SCR*) protein. Here, we examined the function of *S_60_,* one *SP11/SCR* allele of *B. rapa* cv. Osome, using a RNAi-mediated gene silencing approach. The transgenic RNAi lines were highly self-compatible, and this trait was stable in subsequent generations, even after crossing with other commercial lines. These findings also suggested that the resultant self-compatibility could be transferred to commercial cultivars with the desired performances in *B. rapa*.

## Introduction

In flowering plants, self-incompatibility (SI) is a genetic system that promotes outcrossing by rejecting self-related pollen [1]–[3]. In *Brassica*, recognition of pollen is controlled by *S* haplotypes (designated *S_1_*, *S_2_*,... *Sn*), each of which consists of the pollen determinant gene, the S locus protein 11 (*SP11*, also called *SCR*) [4]–[6], and the pistil determinant gene, the S receptor kinase (*SRK*) [7]–[9]. Genes of *SP11/SCR* (*SP11* hereafter) and *SRK* are closely linked each other at the *S* locus [10], and the *S* locus contain multiple alleles [11]–[14]. The self-incompatibility response occurs when the pollen and pistil share the same *S* haplotype. *SP11* interacts with *SRK* of the same *S* haplotype and activates its kinase domain [15]–[17]. This activation is believed to elicit a signaling cascade within the stigmatic papilla cell that leads to rejection of self-pollen [18], [19]. *SP11* is expressed in the anther tapetum, a sporophytic tissue. Therefore, the SI phenotype in pollen is determined by the dominance relationships between the two *S* haplotypes carried by the plant. Based on these relationships, the *S* haplotypes in *Brassica* have been broadly classified into two groups: pollen-dominant *S* haplotypes (class I) and pollen-recessive *S* haplotypes (class II) [14], [20]–[22]. Pollen-dominant *S* haplotypes (such as *S_8_*, *S_9_, S_12_* and *S_52_* in *Brassica rapa*) are generally co-dominant, and are almost always dominant over recessive *S* haplotypes (such as *S_44_, S_60_, S_40_* and *S_29_*) [14], [21]. In *S* heterozygotes with dominant and recessive *S* haplotypes, the expression of the recessive *SP11* allele is silenced as a result of tapetum-specific *de novo* cytosine methylation in its promoter region immediately before the initiation of *SP11* transcription [23], [24]. Additionally, several types of cysteine-rich peptides/polypeptides (CRPs) are expressed specifically in flowers and seeds, where they play reproductive regulatory roles [25]. For instance, *SP11*, with eight conserved cysteines belonging to a subclass of defensin-like proteins, is involved in the inhibition of self-pollen germination and pollen tube growth [15]. Defensin-like *LURE*s act as attractants, guiding pollen tubes to the embryo sac [26], [27].

SI is one of the most important obstructions to *B. rapa* seed production and for that self-compatible (SC) cultivar is very important in case of commercial cultivation. Manipulation of *S* locus genes is one of the most recognized way so far to convert SI into SC in *Brassica*. RNA interference (RNAi)-mediated suppression of stylar 120 kDa glycoprotein (120K), a non-*S*-factor, results in breakdown of the capability of the pistil to reject self-pollen, suggesting that it is required for gametophytic self-incompatibility (GSI) function [28]. In plants, RNAi can be used to regulate endogenous genes [29], and by utilizing a partial sequence of an endogenous gene in the inverted repeat regions of the silencing construct, high-level silencing of the target gene expression can also be achieved. The various RNAi techniques each have advantages and disadvantages with respect to how persistent their effect is and the range of plants to which they can be applied. For example, bombardment can be applied to any plant, but produces only transient effects. Alternatively, transformation with ihpRNA-expressing transgenes provides stable and heritable gene silencing. ihpRNA transgenes have been shown to be very effective for a wide range of target genes in various plant species [30]–[35], indicating that the RNAi mechanism is probably conserved in all plant species.

Here, we report for the first time the use of RNAi gene-silencing constructs to achieve silencing of the *S* locus *SP11* gene *S_60_* resulting in the development of a self-compatible *B. rapa* transgenic line. Furthermore, we demonstrate the stable inheritance of these phenotypes in progeny derived by either selfing or intercrossing and assess the performance of these lines.

## Materials and Methods

### Plant materials and growth conditions

*Brassica rapa* cv. Osome plants were grown under sterile conditions on MS medium during transformation and tissue culture. Transgenic *B. rapa* plants and crossing generations were grown in the greenhouse of the Department of Horticulture, Sunchon National University, Korea, under natural light conditions.

### Preparation of RNAi constructs

**Figure 1:**
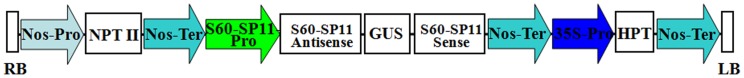
Schematic representations of the S60-SP11RNAi vector construct. A 285 bp cDNA fragment of *S60-SP11* was put into the upstream and downstream of the 1023 bp *Gus* linker of binary vector pBI101 in opposite orientations under control of the S60-SP11 promoter. LB, left border of T-DNA; *NPTII*, gene for neomycin phosphotransferase for kanamycin resistance; 35S-Pro, 35S promoter of cauliflower mosaic virus; Nos-Pro, promoter of nos (nopaline synthase) gene; Nos-Ter, terminator of nos (nopaline synthase) gene; S60-SP11 pro, S60-SP11 promoter; *HPT*, the Hygromycin-resistance gene; RB, right border of T-DNA.

The coding sequence of *S_60_* containing 285 bp nucleotides was placed upstream and downstream of the *Gus* gene encoding the *β*-glucuronidase fragment in opposite directions using the methods described by Chuang and Meyerowitz [36]. This *Gus* gene with the sense and antisense coding sequence of *S_60_* was placed in the *Sal*I and *Sac*I site between the SSH and nopaline synthase terminator of binary vector pBI101 constructed from pBI121 [37]. The SSH fragment was then removed and the *S_60_* promoter of 489 bp was placed in the *Kpn*I and *Sal*I site. The resulting RNAi construct was denoted as S60-SP11RNAi (Figure 1). This construct was then introduced into *Agrobacterium tumefaciens* strain EHA105.

### Plant transformation and regeneration

The hypocotyl transformation protocol developed for *B. rapa* [38], [39] was followed in this study, with some modifications. Briefly, seeds of *B. rapa* cv. Osome were surface-sterilized by washing with 70% ethanol for 2 min, 1% sodium hypochlorite for 15 min and double distilled water for 3–4 times. Seeds were germinated and grown in 0.1× MS medium in a culture room maintained at 22–24°C with a 16 h light/8 h dark photoperiod at a light intensity of 4500–5500 lux. Hypocotyls were excised from 6 to 7-day-old seedlings, cut into segments 2–4 mm in length, and placed onto MS-1 medium and pre-cultured for 24 h under indirect continuous light. Explants were then immersed in a suspension of 1xl0^8^ bacteria/ml for 30 min with shaking at 40 rpm, then returned to feeder plates. After two days of cocultivation with *Agrobacterium*, explants were transferred to B5–1 medium supplemented with 500 mg/l carbenicillin and kept at 24°C under continuous light at 7500 lux intensity for 3–7 days, then transferred to B5-BZ shoot regeneration medium. These explants were cultured for seven days in B5–BZ medium supplemented with 500 mg/l carbenicillin, 10 mg/l Hyg, and 10 mg/l AgNO_3_, followed by 14 days in medium supplemented with 500 mg/l carbenicillin and 20 mg/l Hyg, 14 days in medium supplemented with 500 mg/l carbenicillin and 30 mg/l Hyg and finally, 14 days in medium supplemented with 500 mg/l carbenicillin and 30 mg/l Hyg. The cultures were then transferred onto B5–0 shoot maturation medium supplemented with 500 mg/1 carbenicillin and 50 mg/l Hyg. Two weeks later, shoots were trimmed to contain 2–3 nodes and then placed on B5 root induction medium supplemented with 2 mg/l IBA, 500 mg/l carbenicillin and 50 mg/l Hyg. Roots developed on some of the shoots after two weeks. Shoots that had not rooted were re-cut at the base and placed back onto the medium for another 2–4 weeks.

### DNA extraction and PCR analysis

The putative T_0_, T_1_ and BC_5_F_2_ plants were analyzed by PCR to confirm the presence of transgenes. To accomplish this, plant genomic DNA was isolated from the leaves of each line using the DNeasy Plant Mini Kit (Qiagen, USA). The primers specific for the S60-SP11RNAi cassette (5′- GGC ATA TGA AGC TTG TCG ACA TGA TTT AAC TTT GCA ACAG -3′; 5′- CTG CAG GAG CTC GCG GCC GCA TGA TTT AAC TTT GCA ACAG -3′) and for *NPTII* (5′- CAA GAT GGA TTG CAC GCA GG -3′; 5′- GAA GAA CTC GTC AAG AAG GCG -3′) were used to identify transgenic plants. PCR reactions were carried out in a 20 l mixture at 94°C for 5 min, then subjected to 35 cycles of amplification at 94°C for 1 min, 55°C for 1 min, and 72°C for 2 min. PCR products were visualized by electrophoresis on 1% agarose gel.

### RNA extraction and expression analysis

RNA was extracted from the anthers of T_1_ and BC_5_F_2_ plants using an Rneasy mini kit (Qiagen, USA). RNA was treated with RNase-free DNase (Promega, USA) to remove genomic DNA contaminants. Between 50 and 100 ng of total RNA was used to make single-strand cDNA using SuperScript III reverse transcriptase (Invitrogen, Toronto, Canada) in a 20 µl reaction with oligo (dT18) primers according to the manufacturer's instructions. RT-PCR was conducted using an AMV one step RT-PCR kit (Takara, Japan). Primers specific for *S_60_* (5′- ATG AGA TAT GCT ACT TCT ATA TAT ACA -3′; 5′- TGA TTT AAC TTT GCA ACA GTA GCA -3′) were used for RT-PCR, and actin primers specific for *Brassica* (5′- ATG GCC GAG GCT GAT GAC AT -3′ and 5′- AGC CTC GGT AAG AAG AAC CG -3′) were used as a control. PCR was conducted using 50 ng of cDNA from the anthers of respective plants as templates in master mixes composed of 20 pmol of each primer, 150 µM of each dNTP, 1.2 U of *Taq* polymerase, 1x *Taq* polymerase buffer, and double-distilled H_2_O diluted to a total volume of 20 µl in 0.5 ml PCR tubes. The samples were then subjected to the following conditions: initial denaturation at 94°C for 5 min, followed by 30 cycles of denaturation at 94°C for 30 s, annealing at 58°C for 30 s and extension at 72°C for 1 min, with a final extension for 5 min at 72°C. PCR products were visualized by electrophoresis on 1% agarose gel.

Real-time quantitative PCR was performed using 1 µl of cDNA in a 25 µl reaction employing iTaq^™^ SYBR^®^ Green Super-mix with ROX (California, USA). The same primers used for RT-PCR were employed for real-time PCR, while *Brassica* actin primers (5′- CAA CCA ATC GTC TGT GAC AA -3′; 5′- ATG TCT TGG CCT ACC AAC AA -3′) were used as a control. The conditions for real-time PCR were as follows: initial denaturation for 10 min at 95°C, followed by 40 cycles of 94°C for 30 s, 58°C for 30 s, and 72°C for 45 s. The fluorescence was measured following the last step of each cycle, and three replications were used for each sample. Amplification, detection, and data analysis were conducted using a Rotor-Gene 6000 real-time rotary analyzer (Corbett Life Science, Australia).

### Self-compatibility analysis

SC was tested by observing the pollen-tube behavior and fruit set ratio. For observation of pollen-tube behavior, flower buds were covered with a bag one day before anthesis and then pollinated with pollen from the same plant, after which the samples were placed on 1% solid agar plate at room temperature for 24 h. The pollinated pistils were then separated from the buds and fixed in acetic alcohol (ethanol: acetic acid = 3∶1) for 5 h at room temperature. The fixed pistils were hydrolyzed in 1 N NaOH for 2h at 60 °C, after which they were stained with decolorized aniline blue solution (0.01% aniline blue in 2% K_3_PO_4_) for 2 h. The stained stigmas were mounted on glass slides with 50% glycerol and observed using a UV fluorescent microscope (Nikon Eclipse 80i, Japan). Three flowers were used from each plant. During flowering, the major inflorescence was bagged for self-pollination and the self-compatibility index (SCI) (self-compatibility index  =  the number of seeds/the number of flowers) was calculated according to Zhang et al. [40].

## Results and Discussion

### Identification of target gene and plasmid construction

The sequences of mature SP11 proteins are highly divergent, except for the presence of conserved cysteines [41]. Class II SP11s show several similarities with class I SP11s such as, they are small, secreted proteins with conserved putative signal peptides, their mature proteins show *S*-haplotype-specific polymorphisms in spite of their common hydrophilic and basic properties, like other pollen coat proteins (PCPs), they have eight conserved cysteine residues [20]. All eight cysteine residues are conserved in an arrangement that is characteristic of SP11. Conservation of the eight cysteine residues suggests a common three-dimensional protein structure of SP11s that is stabilized by intramolecular disulfide bonds, similar to the defensin family of antimicrobial proteins [42]. Two other residues that are conserved among most of the class I SP11s, a glycine residue between C1 and C2 and an aromatic amino acid residue between C3 and C4 [41], also are conserved among all of the class II SP11s. Hydrophilicity analysis of the class II SP11s does not suggest a hydrophilic (surface-exposed) structure in the C3–C4 region, which has been suggested for class I SP11s [43]. Three other regions, C1–C2, C2–C3, and C5–C6, are highly divergent and contain two or three amino acid residues that are completely variable across the four class II *S*-haplotypes of *B. rapa* [20]. *B. rapa* cv. Osome, a heterozygote of the *S_52_* and *S_60_* haplotype [44], was used for silencing of *SP11/SCR* gene based on RNA interference. *S_52_* is a class I and *S_60_* is a class II *SP11 S*-haplotype [9], [14]. Amino acid sequence identities among class II SP11s are 62.3 to 94.6%, rather high compared with those of class I SP11s which ranges from 19.5 to 76.1% [20]. Due to having low diversity and number of alleles of class II *SP11s*, we considered class II *SP11*, *S_60_* as a crucial target for conversion of the SI *B. rapa* cv. Osome to SC. The 285 bp-length sequence of the *S_60_* gene was selected as the target for RNA silencing.

We constructed an RNAi binary vector designated as S60-SP11RNAi (Figure 1) for introduction into SI *B. rapa* cv. Osome. The target sequence was incorporated into pBI101 in inverted-repeat orientations interrupted by *GUS* (1023bp). To enhance the efficiency of RNA silencing, strong *S_60_* promoter (−489 to −1) was used to control expression of the transgene, and a plasmid was constructed to specifically suppress expression of *S_60_*. It is well known that the 5′-flanking region up to −192 bp *SP11* is sufficient to direct gene expression in tapetum and pollen [45]. Moreover, the vector carried plant resistant gene *NPTII* driven by the Nos promoter and *HPT* driven by the CaMV 35S promoter.

### Transformation and selection of homozygous transgenic plants

Using the hypocotyl as the explants, plasmid pBI101 with a S60-SP11RNAi cassette was transformed into *B. rapa* mediated by *A. tumefaciens* EHA105. Subsequently, the *NPTII*-resistant plantlets were regenerated, and the insertion of silencing fragments was confirmed by genomic DNA PCR (data not shown). Overall, 16 transgenic plants were obtained and grown to generate T_1_ seeds, after which they were harvested separately. Around, 20 to 25 T_1_ seeds were harvested from each transgenic line by selfing and then cultured in pots to produce T_2_ seeds. For genetic segregation of the T_2_ generation, 15–20 T_2_ seeds from each T_1_ line were tested onto 1/10 strength of MS plate with 50 mg/l hygromycin for antibiotic resistance screening. After five weeks of culture, heterozygote and homozygote events were counted on the basis of hygromycin resistance and susceptibility of plants. Among the 16 transformant lines, three lines produced transformants and non–transformants in a 3∶1 (χ^2^ = 0.003–0.04, P = 0.88–0.93) ratio. This pattern of segregation is only possible when single copy transfer of genes occurs in the transgenic plants. The other transformants were not segregated in such a pattern in the T_2_ generation, indicating the transfer of more than one copy of the target gene. Therefore, only homozygous transformant lines, self-compatible by RNAi (SR) 6–10, SR11-8 and SR18-7 were used for further study.

### Suppression of *S_60_* gene and conversion to self-compatible state

**Figure 2:**
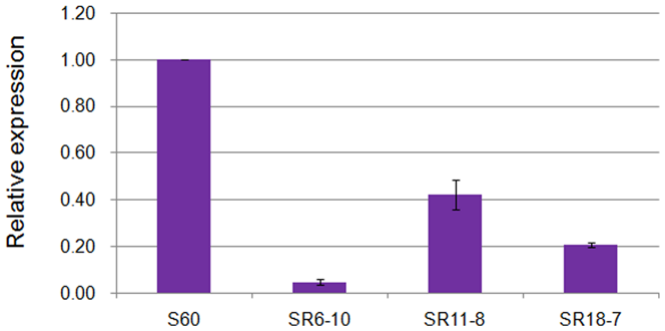
Real-time PCR expression analysis of the *S60-SP11* gene in T_2_ RNAi plants. *S60*: wild type of *S60*, SR6-10, SR11-8 and SR18-7: RNAi transgenic plants.

**Figure 3:**
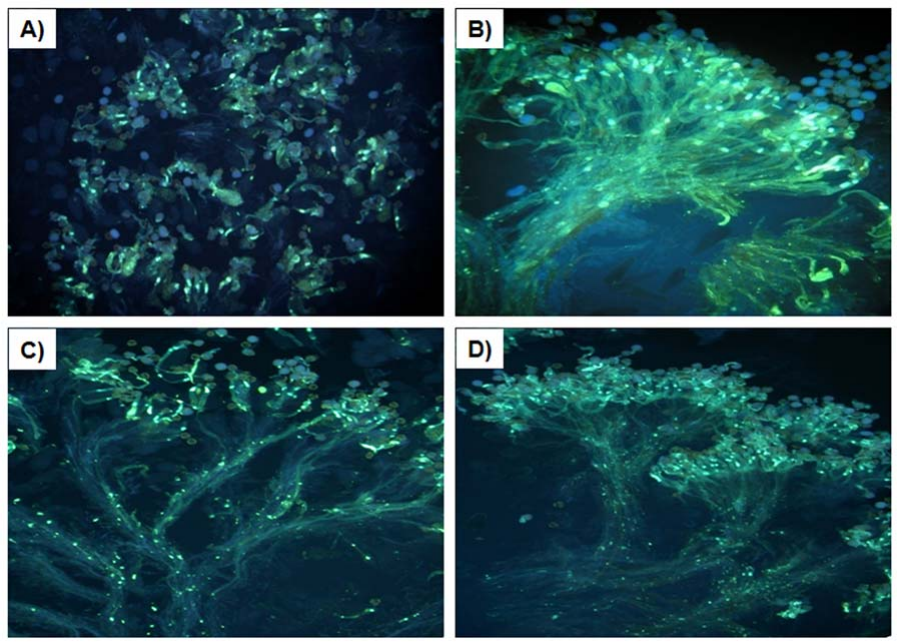
Pollen-tube behaviors of self-pollinated T_2_ generation plants. Photographs were obtained by UV fluorescence microscopy. A): WT, B): SR6-10 RNAi plant, C): SR11-8 RNAi plant, D): SR18-7 RNAi plant.

Real-time PCR was conducted to analyze the relative transcription level of the *S_60_* gene among the three aforementioned homozygotic T_2_ lines. The results revealed much lower transcription levels in the transformants lines than the non-transformant line (Figure 2). Suppression levels varied from almost 2.5 to approximately 20-fold when compared with the untransformed control, with the highest suppression being observed in SR6-10. These three T_2_ lines were self-pollinated to produce T_3_ seeds and the compatibility of these lines was also tested by investigating the pollen tube behavior and fruit set. Unlike the self-incompatible tester line (Figure 3A), the S60-SP11RNAi lines showed numerous pollen tubes on stigma papilla cells upon self-pollination (Figure 3B-D). The mean SCI values of the SR6-10, SR11-8 and SR18-7 T_2_ lines were 6.89, 6.28 and 5.91, respectively, which were comparable to their average seed per open-pollinated flower values (7.95, 7.55 and 7.38). The mean SCI of the tester line was 0.82. Taken together, the pollen tube observations and fruit set data confirmed that the S60-SP11RNAi lines were highly self-compatible.

The SI system is sporophytic in *Brassica*, and *SP11*s from class-II *S*-haplotypes (e.g. *S_40_* and *S_60_*) exhibited strictly sporophytic expression patterns, suggesting that the expression of *SP11* in the tapetal cell layer was sufficient for SI [20]. Recent biochemical studies revealed that *SP11* functions as the sole ligand for its cognate *SRK* receptor complex. Their interaction induces the autophosphorylation of *SRK*, which is expected to trigger the signaling cascade that results in the rejection of self-pollen. This so-called ligand receptor complex interaction and receptor activation occurs in an *S*-haplotype-specific manner, and this specificity is almost certainly the basis for self-pollen recognition [5], [15]. In this study, we silenced the *S_60_* gene in *B. rapa* to interfere with the so-called ligand receptor interaction and receptor activation and prevent consequent rejection of self-pollen and the resultant S60-SP11RNAi homozygous plants were SC.

### Stable inheritance of the trait

**Figure 4:**
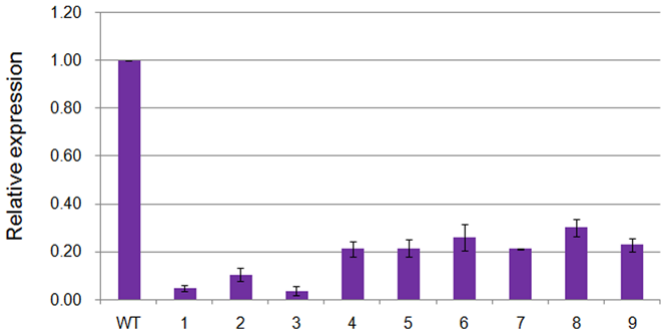
Real-time PCR expression analysis of the *S60-SP11* gene in T3 generation plants. WT: wild type of *S60*, lane 1–3: SR6-10 RNAi plants, lane 4–6: SR11-8 RNAi plants and lane 7–9: SR18-7 RNAi plants.

To determine the stability of gene silencing in the following generations, three T_2_ lines that had relatively strong gene silencing based on the reduction of the transcript abundance levels were selected for further analysis. All T_3_ individuals produced from each T_2_ line were checked through amplification of *NPTII* and *S_60_* primers using gDNA extracted from 4-week-old leaves to identify positive and negative progenies and all plants were found to be positive (data not shown). *S_60_* gene expression in these plants was also analyzed through RT-PCR analysis of three plants selected at random from each T_3_ line using *S_60_* specific primers (Figure 4). The results showed that the gene suppression level was similar to that of T_1_ transgenic plants when compared to the untransformed control.

### Performance evaluation of RNAi transgenic lines

Transgenic SC *B. rapa* lines were subjected to performance evaluation as seed set experiments under two different conditions; use of pollinating agents (Bees and CO_2_) and no pollinating agents. It should be noted that the commercial cultivation of different *Brassica* crops uses bees and CO_2_ as pollinating agents, and CO_2_ is not environment friendly. In this evaluation study, three homozygous RNAi transgenic lines and a non-transformant (WT) line were used. For the non-transformant plants, bees and CO_2_ were used as pollinating agents, while for the transformant plants no bees or CO_2_ were used (File S1). In each case, the total seed number was counted from four plants and highest average seed set was found in the SR6-10 RNAi transgenic line (Table 1). These findings indicate that the transgenic RNAi lines could perform well in fruit settings without any pollinating agents and could be used for commercial cultivation with great economic benefits and minimal environmental hazards.

**Table 1:** Comparative seed production in non-transgenic control and S60-SP11RNAi plants of T_3_ generation.

Name of lines	No. of plants used	Total amount of Seed produced (g)	Average seed weight/plant (g)	Ways of pollination
Non-transgenic	4	170	42.5	CO_2_ and Bees
SR6-10	4	280	70	Without CO_2_ and Bees
SR11-8	4	115	28.75	
SR18-7	4	30	7.5	

### Use of RNAi transgenic line as breeding material

**Figure 5:**
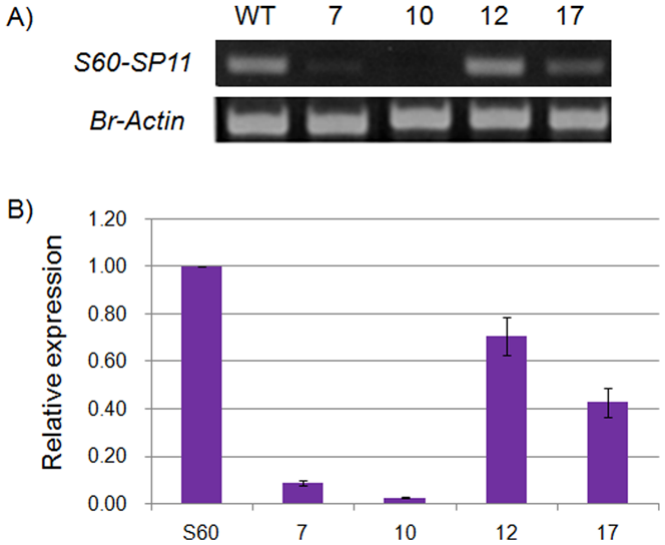
Expression analysis of *S60-SP11RNAi* gene in BC_5_F_2_ generation plants using *S60-SP11* specific primers and *Br-Actin* primers were used as control. A) RT-PCR and B) Real-time PCR expression analysis. WT: wild type of *S_60_*; 7, 10, 12 and 17: Homozygous lines of BC_5_F_2_ generation after backcrossing between SR6-10 transgenic plant and *B. rapa* ‘Seoulbechhu’ plant.

SI is generally the main obstruction for *B. rapa* seed production and for that SC cultivar is crucial issue in case of commercial seed production. In this study, we developed a SC *B. rapa* line and then transferred this trait in a strictly SI commercial variety, *B. rapa* ‘Seoulbechhu’. We utilized the backcross method to transfer the *S60-SP11RNAi* gene from the SR6-10 RNAi transgenic homozygous line into this variety and obtained 19 lines in the BC_5_F_2_ generation. The insertion of silencing fragments was confirmed by genomic DNA PCR of these 19 lines using hygromycin resistant gene primers (data not shown). We then re-checked the inserts using class I and class II *SRK* universal primers to identify the homozygous lines for the *S_60_* gene introduced into these 19 lines and found no class I insert in lines no. 7, 10, 12 and 17 (File S2), indicating that these four lines are homozygous for class II *S60-SP11RNAi* gene. Because pollen-dominant class I *S* haplotypes (such as *S_8_, S_9_, S_12_* and *S_52_* in *Brassica rapa*) are generally co-dominant, they are almost always dominant over recessive class II *S* haplotypes (such as *S_44_, S_60_, S_40_* and *S_29_*) [14], [21]. Upon expression analysis, only these four lines showed expression of the *S60-SP11RNAi* gene at different levels, while no expression was observed in the other lines (File S3). The expression level of the *S60-SP11RNAi* gene in these four homozygous lines was compared with that of non-transformed *S_60_* containing plants. Line 10 showed the highest level of suppression when compared to non-transformed *S_60_* bearing plants, followed by lines 7, 17 and 12 (Figure 5). The fruit setting of these four lines was also very high in this generation.

Taken together, it can be concluded that the transgenic RNAi lines were fully self-compatible, and that this trait was stable in subsequent generations, even after crossing with other commercial lines with higher performance than the non-transgenic lines.

## Supporting Information

File S1 Fruit set analysis in S60-SP11RNAi plants of T_3_ generation. A) Non-transgenic control (with CO_2_ & Bees), B) SR6-10 lines (without CO_2_ & Bees), C) SR11-8 lines (without CO_2_ & Bees), D) SR18-7 lines (without CO_2_ & Bees).
(TIF)

File S2 Genomic DNA PCR analysis of 19 BC_5_F_2_ (backcrossing between SR6-10 transgenic plant and *B. rapa* ‘Seoulbechhu’ plant) generation plants using *SRK,* A) class I and B) class II universal primers.
(TIF)

File S3 RT-PCR expression analysis of S60-SP11RNAi gene in 19 BC_5_F_2_ generation plants using *S60-SP11* specific primers and *Br-Actin* primers were used as control. WT: wild type of *S_60_*, 1–19: lines of BC_5_F_2_ generation.
(TIF)
