# Supplementary figures and images for: Development of Self-Compatible B. rapa by RNAi-Mediated S Locus Gene Silencing
Source: PLoS One. 2012 Nov 8;7(11):e49497. doi: 10.1371/journal.pone.0049497 (PMC3493532; doi:10.1371/journal.pone.0049497)

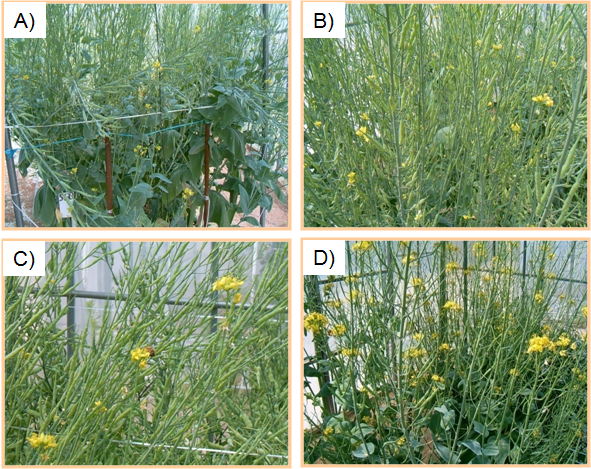

Supplement: File S1 — Fruit set analysis in S60-SP11RNAi plants of T3 generation. A) Non-transgenic control (with CO2 & Bees), B) SR6-10 lines (without CO2 & Bees), C) SR11-8 lines (without CO2 & Bees), D) SR18-7 lines (without CO2 & Bees). (TIF) [file pone.0049497.s001.tif]

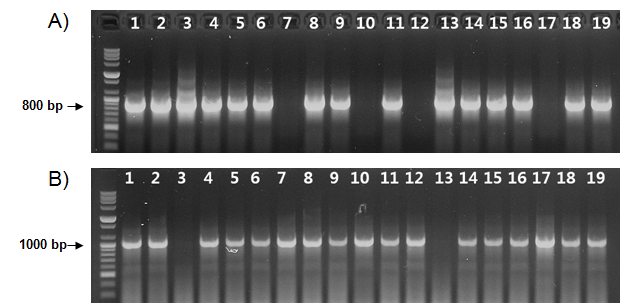

Supplement: File S2 — Genomic DNA PCR analysis of 19 BC5F2 (backcrossing between SR6-10 transgenic plant and B. rapa ‘Seoulbechhu’ plant) generation plants using SRK, A) class I and B) class II universal primers. (TIF) [file pone.0049497.s002.tif]

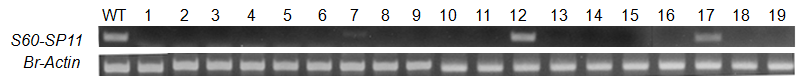

Supplement: File S3 — RT-PCR expression analysis of S60-SP11RNAi gene in 19 BC5F2 generation plants using S60-SP11 specific primers and Br-Actin primers were used as control. WT: wild type of S60, 1–19: lines of BC5F2 generation. (TIF) [file pone.0049497.s003.tif]
